# Concentrations and temporal trends in pesticide biomarkers in urine of Swedish adolescents, 2000–2017

**DOI:** 10.1038/s41370-020-0212-8

**Published:** 2020-02-24

**Authors:** Erika Norén, Christian Lindh, Lars Rylander, Anders Glynn, Jonatan Axelsson, Margareta Littorin, Moosa Faniband, Estelle Larsson, Christel Nielsen

**Affiliations:** 1 Division of Occupational and Environmental Medicine, Department of Laboratory Medicine, Lund University, Lund, Sweden; 2 Department of Biomedical Sciences and Veterinary Public Health, Swedish University of Agricultural Sciences (SLU), Uppsala, Sweden

**Keywords:** Biomonitoring, Dietary exposure, Pesticides, Population based studies

## Abstract

Agricultural pesticides are extensively used for weed- and pest control, resulting in residues of these compounds in food. The general population is mainly exposed through dietary intake. Exposure to certain pesticides has been associated with adverse human health outcomes. Our aim was to assess urinary concentrations and temporal trends in the biomarkers of commonly used pesticides. Samples were collected from adolescents (*n* = 1060) in Scania, Sweden, from 2000 to 2017. Concentrations of 14 pesticide biomarkers were analyzed in urine using LC–MS/MS. Temporal trends in biomarker concentrations (ln-transformed) were evaluated using linear regression. Biomarkers of pyrethroids (3-PBA and DCCA), chlorpyrifos (TCPy), chlormequat (CCC), thiabendazole (OH-TBZ), and mancozeb (ETU) were detected in >90% of the population all sampling years. The biomarkers CCC and TCPy had the highest median concentrations (>0.8 µg/L), whereas the biomarkers of cyfluthrin (4F-3-PBA) and two pyrethroids (CFCA) had the lowest median concentrations (<0.02 µg/L). Increasing temporal trends were found for the biomarkers 3-PBA (3.7%/year), TCPy (1.7%/year) and biomarkers of pyrimethanil (11.9%/year) and tebuconazole (12.2%/year). Decreasing trends were found for CCC (–5.5%/year), OH-TBZ (−5.5%/year), and ETU (−3.9%/year). Our results suggest that Swedish adolescents are commonly exposed to pesticides in low concentrations (median concentrations <3.88 µg/L).

## Introduction

Concentrations of chemical substances in human biological samples reflect the total environmental exposure from our surroundings. Agricultural pesticides are a broad group of chemicals extensively used over the last century to control pests, including weeds. They are designed to target specific pests, an advantage for agricultural production, but they could also be potentially toxic to the environment, animals, or humans. The wide application of pesticides has led to residues of these compounds in food products [1–3]. The general population may therefore be continuously exposed through diet, mainly from intake of vegetables, fruits, and grains [4].

Contemporary pesticides are designed to be nonpersistent and less toxic than older, currently replaced or restricted, pesticides [5]. Despite this, they are still suspected to be associated with adverse human health outcomes mainly through occupational exposure but sometimes through indirect exposure in the general population [6]. The toxicities of several contemporary pesticides, such as thiabendazole, mancozeb, and chlorpyrifos, have been evaluated in animal studies confirming neuro- and hepatotoxicity and endocrine disruption [7–9]. Human exposure and health effects have mainly been studied for e.g., pyrethroid and organophosphate pesticides and phenoxy herbicides [10]. Early life exposure has been reported to be associated with human health effects on neurodevelopment and cognitive function in the general population and in children living near agricultural areas [11, 12]. However, there is no or very limited knowledge of many other groups of pesticides, including certain fungicide groups and growth regulators.

Biomonitoring is a way to observe the level of pesticide exposure in human populations and is an important part of evaluating existing pesticide regulations and restrictions [13–15]. Currently used pesticides generally have short biological half-lives and are rapidly excreted in urine. Measurement of urinary biomarkers is an established method to study exposure and reflects all exposure pathways [16].

Occupational exposure to pesticides in current use, such as organophosphate insecticides and a few fungicides, has been monitored in agricultural workers in several countries [17–19]. Some major general population biomonitoring studies on pesticides are conducted through the National Health and Nutrition Examination Survey in the USA [20–24], the German Environmental Survey [25], and the Canadian Health Measures Survey [3, 26]. There are also studies of organophosphate and pyrethroid insecticides, dithiocarbamates, chlormequat, azole compounds, and phenoxy herbicides in groups from the general populations in, for example, France [27], Israel [28], the UK [29, 30], Italy [31], Germany [32, 33], and Australia [34].

Exposure surveillance over time, preferably by biomonitoring, is considered an important component of exposure assessment. Temporal trend studies of exposure can be used to follow-up the effects of risk-limiting measures and regulation and to identify exposure to emerging new pesticides. However, studies of temporal trends in pesticide exposure are very rare [23]. For several pesticides we still have limited or no knowledge of exposure biomarker concentrations in the general population, exposure variability over time and whether there are associations between exposure and human health.

The objective of this study was to assess exposure levels and potential temporal trends, 2000–2017, of common currently used insecticides, herbicides, fungicides, and growth regulators by analyzing their biomarkers (*n* = 14) in urine from Swedish adolescents in the general population. The pesticides we chose to analyze were selected mainly because of their frequent use globally and their detection in food products in Sweden [4, 35, 36]. Furthermore, some of the compounds were included because they have rarely been monitored in general populations.

## Materials and methods

### Sample collection

Study participants aged 17–21 years were recruited using a cross-sectional study design with samplings at five occasions. All samplings consisted of one random spot urine sample per study participant. In the first 3 sample collection years (i.e., 2000, 2004, and 2009), the population comprised males recruited through the enrollment process for military service in the county of Scania, Sweden [37] (*n* = 271, 200, and 314, respectively). The 2009 cohort also included friends and classmates of the study participants. Information about the study was given to all men in Scania enrolled in the military entrance assessment at the time of recruitment. They were then offered to participate in the study at the same occasion as their military entrance assessment. Spot urine samples were portioned with 1–2 mL urine in 10 mL plastic vials at the sampling location. The sample collections in 2013 and 2017 took place after the abolishment of mandatory military service in Sweden in 2010. We contacted principals at secondary schools in Scania within a 60 km radius from Malmö and three schools accepted to participate, two in Lund and one in Trelleborg. Students in their final year were given oral and written information about the study at their schools and could thereafter volunteer to participate during the same week at their schools. We aimed for 200 study participants per sampling year based on the population size in previous recruitments year 2000–2009 (Table 1). We recruited 204 students in year 2013, out of which 97 participants were males, and 196 students in 2017, out of which 88 participants were males. Sampling material was handed out to the participants at the schools and the urine samples were delivered in a 10 mL plastic vial to field personnel. All the study participants delivered one random spot urine sample and reported information regarding sex, weight, height, age, and smoking habits. Samples were stored at –20 °C in the sampling vials until analysis. The study was approved by the regional ethical review board in Lund (ref. no. 2018/139) and all study participants signed an informed written consent.

**Table 1:** Population characteristics.

Age (years)	Sampling year	*n* (missing)	Median	P25–P75
	2000	271 (0)	18	18–18
	2004	200 (0)	18	18–18
	2009	314 (0)	18	18–18
	2013	199 (5)	18	18–18
	2017	195 (1)	18	17–18

BMI		*n* (missing)	Median	P25–P75
	2000	269 (2)	22.1	20.7–23.5
	2004	200 (0)	22.2	20.4–23.8
	2009	314 (0)	22.7	21.1–24.8
	2013	195 (9)	22.0	20.2–24.7
	2017	195 (1)	22.1	20.3–23.7

Smoking status		*n* (missing)	Yes (*n*)	% Smokers
	2000	270 (1)	73	27
	2004	192 (8)	19	9.9
	2009	314 (0)	64	20
	2013	204 (0)	142	30
	2017	196 (0)	13	6.6

Sex		*n* (missing)	Males	% Males
	2000	271 (0)	271	100
	2004	200 (0)	200	100
	2009	314 (0)	314	100
	2013	204 (0)	97	47.5
	2017	196 (0)	88	44.9

Nonparametric descriptive statistics of study participants, expressed per sampling year.

### Analysis

The analyses were performed using a liquid chromatography system (UFLC^RX^; Shimadzu Corporation, Kyoto, Japan) coupled with triple quadrupole linear ion trap mass spectrometer equipped with TurboIonSpray source (QTRAP 5500 and 6500+; AB Sciex, Foster City, CA, USA). The analyzed exposure biomarkers of the parent pesticides were 3-phenoxybenzoic acid (3-PBA), 4-fluoro-3-phenoxybenzoic acid (4F-3-PBA), 3-(2-chloro-3,3,3-trifluoroprop-1-enyl)-2,2-dimethylcyclopropanecarboxylic acid (CFCA), 3-(2,2-dichlorovinyl)-2,2-dimethylcyclopropanecarboxylic acid (DCCA), 3,5,6-trichloro-2-pyridinol (TCPy), chlormequat (CCC), mepiquat (MQ), 4-hydroxypyrimethanil (OH-PYM), 5-hydroxythiabendazole (OH-TBZ), hydroxy tebuconazole (OH-TEB), ethylene thiourea (ETU), propylene thiourea (PTU), 2,4-dichlorophenoxyacetic acid (2,4-D), and 2-methyl-4-dichlorophenoxyacetic acid (MCPA), The parent compounds and their corresponding biomarkers are shown in Table 2, with their full chemical names shown in Supplementary material (Supplementary I, Table A). Modified methods, including the transitions of the compounds and internal standards, are described and shown in the Supplementary material (Supplementary II—V, Table B).

**Table 2:** Pesticide compounds and corresponding exposure biomarkers.

Pesticide category	Parent compound	Biomarker	Abbrev	LOD	QC mean	QC CV
Insecticide	Pyrethroids^a^	3-Phenoxybenzoic acid	3-PBA	0.009	20.5	10
	Cyfluthrin	4-Fluoro-3-phenoxybenzoic acid	4F-3-PBA	0.005	20.9	6.0
	Bifenthrin λ-cyhalothrin	3-(-2-Chloro-3,3,3-trifluoroprop-1-enyl)-2,2-dimethylcyclopropanecarboxylic acid	CFCA	0.006	21.8	8.1
	Permethrin cypermethrin cyfluthrin	3-(2,2-Dichlorovinyl)-2,2-dimethylcyclopropanecarboxylic acid	DCCA	0.017	22.7	9.8
	Chlorpyrifos	3,5,6-Trichloro-2-pyridinol	TCPy	0.063	23.5	4.9
Growth regulator	Chlormequat	Chlormequat	CCC	0.010	31.3	4.7
	Mepiquat	Mepiquat	MQ	0.011	8.40	5.1
Fungicide	Pyrimethanil	4-Hydroxy-pyrimethanil	OH-PYM	0.004	21.7	7.3
	Thiabendazole	5-Hydroxy-thiabendazole	OH-TBZ	0.002	21.1	3.9
	Tebuconazole	Hydroxy tebuconazole	OH-TEB	0.016	20.7	4.2
	EBDCs^b^	Ethylene thiourea	ETU	0.016	24.2	3.1
	Propineb	Propylene thiourea	PTU	0.1	21.3	22
Herbicide	2,4-D	2,4-Dichlorophenoxyacetic acid	2,4-D	0.108	22.2	6.8
	MCPA	2-Methyl-4-dichlorophenoxyacetic acid	MCPA	0.016	21.9	4.5

List of parent compounds and their corresponding biomarkers analyzed in urine. Calculated limit of detection (LOD) is listed in µg/L. Mean (µg/L) and coefficient of variance, CV (%), for quality control (QC) samples are listed for each compound.

^a^Nonspecific biomarker of the pyrethroids permethrin, cypermethrin, deltamethrin, resmethrin, fenvalerate, phenothrin, cyphenothrin and lambda-cyhalothrin.

^b^Nonspecific biomarker of several ethylene bis-dithiocarbamate (EBDC) fungicides but most commonly for mancozeb.

A modified method based on Faniband et al. [38] was applied for the simultaneous analysis of 3-PBA, 4F-3-PBA, CFCA, DCCA, TCPy, OH-PYM, OH-TBZ, OH-TEB, 2,4-D, and MCPA. In short, urine samples were enzymatically treated overnight with β-glucuronidase/arylsulfatase and extracted on 96-well solid phase extraction (SPE) plates before LC–MS/MS analysis. The ETU analysis was performed using a modified method described by Ekman et al. [39] that was further used after modification for the analysis of PTU. In short, urine samples were hydrolyzed with NaOH and incubated at 100 °C for 1 h before analysis. The analyses of MQ and CCC were performed using a modified method described by Lindh et al. [40]. Briefly, urine samples were diluted and extracted using SPE in 96-well format before analysis. In each analysis, the sample plates contained calibration standards, chemical blanks prepared from Milli-Q water, and quality control (QC) samples prepared from blank urine or authentic urine samples spiked with the analyte. Previously unpublished details of the analytical methods are further described in the Supplementary material. All samples were analyzed together (randomized), except samples from 2017, at the laboratory of the Division of Occupational and Environmental Medicine in Lund, Sweden. The laboratory takes part in the Erlangen interlaboratory program for TCPy and 3-PBA with excellent results.

We measured the urinary density using a hand refractometer. The limit of detection (LOD) was defined as three times the standard deviation of the concentration corresponding to the peak-area ratio in the chemical blanks. The mean value of the chemical blanks from all batches (*n* = 14) of samples was used to estimate the LOD for each biomarker. The between-run precision of the method was determined using the QC samples and is presented as the mean value and coefficient of variation (CV) in Table 2. Urinary creatinine was determined using an enzymatic method [41].

### Statistical analysis

Negative concentrations, a consequence of subtracting the chemical blanks, were replaced with the lowest measured positive concentration <LOD [42] divided by $\sqrt {2}$, a constant usually used for imputing observations below LOD [43]. Positive values below LOD were used in the statistical analyses without imputation to maintain the variability in the material that could affect observations of possible trends [44]. All measured concentrations were adjusted for density using calculations described elsewhere [45], and adjusted for creatinine. Statistical analysis was performed using IBM SPSS software (version 24.0). Descriptive statistics of population characteristics and urinary concentrations were presented through nonparametric statistics because the data were not normally distributed.

Trends over time were initially explored using boxplots of biomarker concentrations plotted against calendar year. Our data followed a log-normal distribution and was ln-transformed to achieve normality. Temporal trends were formally evaluated using linear regression, including all observations, by regressing ln-transformed concentrations on calendar year (continuous variable). Quadratic terms were explored when the boxplot indicated that the relationship might not be linear. Model validation was conducted through residual analysis using QQ plots and models that failed to meet the assumptions of linear regression were excluded from the temporal trend analysis. The coefficient of determination (*R*^2^) was calculated to assess the amount of variance in the biomarker concentrations that was explained by calendar year. The robustness of the regression models was assessed through sensitivity analyses (1) excluding values above the 95th percentile and (2) excluding female participants.

## Results

### Urinary biomarker concentrations

**Fig. 1:**
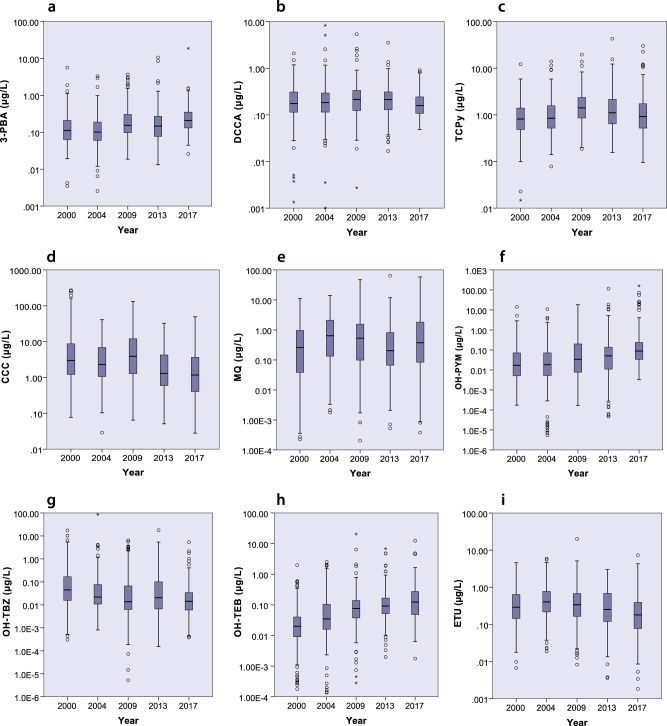
Urinary concentrations of pesticide biomarkers. Density-adjusted concentrations in urine (log-scale) for metabolites: **a** 3-PBA, **b** DCCA, **c** TCPy, **d** CCC, **e** MQ, **f** OH-TBZ, **g** OH-PYM, **h** OH-TEB, and **i** ETU in Swedish adolescents in Scania plotted against sampling year.

The overall highest median concentrations were observed for CCC followed by TCPy (Table 3). The lowest or undetectable median concentrations were observed for CFCA and 4F-3-PBA. The biomarkers 3-PBA, DCCA, TCPy, OH-TBZ, ETU, CCC, and MQ were found in concentrations above LOD in more than 90% of the population all sampling years. The remaining biomarkers 4F-3-PBA, CFCA, OH-PYM, OH-TEB, 2,4-D, and MCPA were all detected in concentrations above LOD in more than 40% of the study population. The biomarker PTU had concentrations below LOD in 95% of all the samples and the results are therefore not reported. Boxplots of biomarker concentrations versus sampling year are shown in Fig. 1a–i. Characteristics of the population (i.e., age, sex, BMI, and smoking habits) are listed in Table 1 for each sampling year. Median BMI was similar all years (20.2–21.1) and smoking status varied from 7 to 30% between different years.

**Table 3:** Descriptive statistics of urine concentrations.

Compound	Year	*n*	% >LOD	Median	P25–P75	P95	Maximum
3-PBA	2000	209	99	0.11 (0.08)	0.06–0.22 (0.05–0.14)	0.62 (0.43)	5.64 (3.43)
	2004	197	96	0.10 (0.09)	0.06–0.19 (0.05–0.16)	0.56 (0.50)	3.29 (2.66)
	2009	254	100	0.15 (0.12)	0.10–0.30 (0.08–0.24)	1.02 (0.90)	3.68 (2.79)
	2013	294	100	0.15 (0.11)	0.08–0.27 (0.06–0.20)	0.79 (0.62)	10.6 (7.89)
	2017	195	100	0.21 (0.15)	0.13–0.35 (0.11–0.30)	0.92 (0.65)	18.8 (15.2)
4F-3-PBA	2000	209	63	0.01 (<LOD)	<LOD—0.01 (<LOD—0.01)	0.03 (0.03)	0.38 (0.21)
	2004	197	54	0.01 (<LOD)	<LOD—0.01 (<LOD—0.01)	0.02 (0.02)	0.05 (0.06)
	2009	254	74	0.01 (<LOD)	<LOD—0.01 (<LOD—0.01)	0.03 (0.03)	0.25 (0.22)
	2013	204	62	0.01 (<LOD)	<LOD—0.01 (<LOD—0.01)	0.02 (0.02)	0.29 (0.44)
	2017	195	42	<LOD (<LOD)	<LOD—0.01 (<LOD—0.01)	0.06 (0.04)	0.76 (0.54)
CFCA	2000	209	39	<LOD (<LOD)	<LOD—0.01 (<LOD—0.01)	0.05 (0.03)	0.35 (0.18)
	2004	197	52	0.01 (0.01)	<LOD—0.01 (<LOD—0.01)	0.03 (0.04)	0.09 (0.11)
	2009	254	76	0.01 (0.01)	<LOD—0.03 (<LOD—0.02)	0.10 (0.07)	1.72 (0.90)
	2013	204	86	0.02 (0.02)	0.01–0.04 (0.01–0.03)	0.09 (0.07)	0.38 (0.39)
	2017	195	90	0.02 (0.02)	0.01–0.05 (0.01–0.04)	0.40 (0.31)	1.76 (1.20)
DCCA	2000	209	97	0.18 (0.13)	0.11–0.31 (0.08–0.21)	0.78 (0.45)	2.08 (3.09)
	2004	197	98	0.18 (0.18)	0.12–0.30 (0.10–0.27)	0.89 (0.86)	8.28 (7.47)
	2009	254	99	0.22 (0.16)	0.12–0.34 (0.10–0.25)	0.79 (0.60)	5.35 (3.23)
	2013	204	99	0.21 (0.15)	0.13–0.31 (0.10–0.24)	0.63 (0.49)	3.53 (2.06)
	2017	195	99	0.16 (0.13)	0.11–0.25 (0.09–0.20)	0.53 (0.41)	0.91 (1.20)
TCPy	2000	209	99	0.82 (0.59)	0.48–1.40 (0.38–0.96)	3.50 (2.38)	12.2 (7.39)
	2004	197	99	0.84 (0.80)	0.53–1.60 (0.48–1.47)	4.01 (3.69)	13.9 (10.1)
	2009	254	100	1.41 (1.08)	0.85–2.37 (0.68–1.80)	5.21 (3.99)	19.6 (11.4)
	2013	204	100	1.11 (0.86)	0.64–2.16 (0.51–1.60)	5.23 (5.06)	43.0 (37.6)
	2017	195	99	0.92 (0.70)	0.52–1.74 (0.46–1.36)	6.54 (5.19)	30.1 (16.6)
CCC	2000	196	100	2.98 (2.01)	1.21–8.71 (0.75–6.64)	139 (66.1)	272 (405)
	2004	197	100	2.30 (2.09)	1.05–6.88 (6.94–15.5)	16.4 (15.5)	40.9 (40.3)
	2009	254	100	3.88 (2.77)	1.28–12.1 (0.89–10.4)	41.5 (30.6)	132 (132)
	2013	204	100	1.29 (0.98)	0.59–4.25 (0.41–3.80)	17.1 (16.2)	32.5 (25.1)
	2017	196	100	1.17 (0.86)	0.40–3.66 (0.31–3.18)	16.8 (13.6)	49.0 (35.8)
MQ	2000	196	87	0.26 (0.16)	0.04–0.97 (0.03–0.73)	3.80 (2.94)	11.2 (16.6)
	2004	197	96	0.64 (0.52)	0.13–2.11 (0.12–2.01)	6.18 (6.40)	14.0 (19.1)
	2009	254	97	0.53 (0.33)	0.10–1.56 (0.08–1.21)	6.19 (5.10)	47.3 (42.8)
	2013	204	95	0.21 (0.17)	0.07–0.83 (0.05–0.72)	3.75 (3.02)	64.1 (62.5)
	2017	196	96	0.37 (0.31)	0.08–1.83 (0.07–1.50)	7.58 (6.02)	58.3 (30.1)
OH-TBZ	2000	209	98	0.05 (0.03)	0.02–0.17 (0.01–0.09)	1.99 (1.91)	17.4 (13.7)
	2004	197	95	0.02 (0.02)	0.01–0.08 (0.01–0.08)	0.92 (0.80)	87.1 (53.7)
	2009	254	97	0.01 (0.01)	0.01–0.07 (0.00–0.06)	1.60 (1.41)	6.41 (5.20)
	2013	204	92	0.02 (0.02)	0.01–0.10 (0.00–0.09)	1.83 (1.27)	17.8 (21.1)
	2017	195	94	0.01 (0.09)	0.01–0.04 (0.00–0.03)	0.40 (0.31)	5.37 (4.51)
OH-PYM	2000	209	76	0.02 (0.01)	0.01–0.07 (<LOD—0.05)	0.42 (0.29)	14.3 (8.67)
	2004	197	79	0.02 (0.02)	0.01–0.07 (<LOD—0.07)	0.96 (0.96)	10.8 (6.84)
	2009	254	88	0.03 (0.02)	0.01–0.20 (0.00–0.15)	1.47 (1.30)	18.2 (22.9)
	2013	204	93	0.05 (0.04)	0.01–0.14 (0.01–0.11)	1.63 (1.24)	116 (98.2)
	2017	195	99	0.09 (0.07)	0.03–0.24 (0.03–0.21)	18.0 (13.7)	161 (136)
OH-TEB	2000	209	62	0.02 (0.01)	<LOD—0.04 (0.01–0.03)	0.31 (0.22)	1.98 (0.68)
	2004	197	76	0.04 (0.03)	0.02–0.11 (0.02–0.09)	0.78 (0.85)	2.54 (2.39)
	2009	254	93	0.08 (0.06)	0.04–0.14 (0.03–0.10)	0.38 (0.34)	20.5 (12.0)
	2013	204	93	0.09 (0.08)	0.05–0.17 (0.04–0.14)	0.90 (0.71)	6.87 (3.66)
	2017	195	94	0.12 (0.09)	0.05–0.27 (0.04–0.22)	1.03 (0.86)	12.4 (14.7)
ETU	2000	149	99	0.29 (0.22)	0.14–0.64 (0.10–0.45)	1.68 (1.10)	4.58 (4.40)
	2004	196	99	0.40 (0.38)	0.22–0.77 (0.19–0.76)	3.03 (2.87)	5.89 (7–26)
	2009	254	99	0.34 (0.26)	0.16–0.68 (0.13–0.52)	2.02 (1.54)	20.0 (8.84)
	2013	204	97	0.25 (0.22)	0.12–0.69 (0.09–0.46)	1.60 (1.26)	3.02 (5.23)
	2017	196	98	0.18 (0.15)	0.08–0.39 (0.06–0.34)	1.19 (1.02)	7.30 (6.80)
2,4-D	2000	209	65	0.12 (<LOD)	<LOD—0.20 (<LOD—0.14)	0.69 (0.48)	2.26 (2.14)
	2004	197	58	0.12 (0.11)	<LOD—0.21 (<LOD—0.20)	0.44 (0.40)	0.95 (0.82)
	2009	254	58	<LOD (<LOD)	<LOD—0.15 (<LOD—0.11)	0.35 (0.24)	2.05 (2.13)
	2013	204	69	0.13 (0.10)	<LOD—0.22 (<LOD—0.17)	0.43 (0.43)	2.24 (0.39)
	2017	195	39	<LOD (<LOD)	<LOD—0.14 (<LOD—0.11)	0.37 (0.29)	1.64 (1.28)
MCPA	2000	209	72	0.03 (0.02)	<LOD—0.05 (<LOD—0.03)	0.12 (0.11)	1.06 (0.56)
	2004	197	73	0.03 (0.03)	0.02–0.05 (0.02–0.05)	0.17 (0.14)	0.90 (0.75)
	2009	254	69	0.02 (0.02)	<LOD—0.04 (<LOD—0.03)	0.14 (0.11)	0.41 (0.40)
	2013	204	66	0.02 (0.02)	<LOD—0.05 (<LOD—0.04)	0.16 (0.13)	0.63 (0.67)
	2017	195	55	0.02 (0.01)	<LOD—0.03 (<LOD—0.02)	0.10 (0.07)	1.78 (1.22)

Nonparametric estimates of density-adjusted concentrations in urine samples (µg/L). Creatinine adjusted concentrations are presented in brackets (µg/g creatinine). Detection frequency is reported as percent above limit of detection.

### Temporal trends in urinary biomarkers

Biomarkers that met the assumptions for linearity, after ln-transformation, and could be evaluated using linear regression are shown in Fig. 1a–i. Increasing trends were observed for the biomarkers OH-PYM, OH-TEB, TCPy, and 3-PBA between 2000 and 2017 (Table 4). The biomarkers OH-PYM and OH-TEB had the highest annual changes in concentrations (>10% per year), though their overall concentrations were still lower than those of the other studied biomarkers. The increasing trends were still observed after the sensitivity analyses. The regression model indicated an increasing trend for MQ, but the model estimates varied in the sensitivity analyses. Decreasing trends were seen for OH-TBZ, ETU, and CCC but with a low annual change in concentration (average 5% per year). No trends were observed for the remaining biomarkers included in the regression models (Table 4).

**Table 4:** Analysis of temporal trends through linear regression.

	All samples			
Biomarker	*β*	95 CI (%)	*p*	*R*^2^
3-PBA	3.7	2.6, 4.7	<0.01	0.05
DCCA	–0.1	–1.0, –0.8	0.802	0.00
TCPy	1.7	0.8, 2.6	<0.01	0.01
CCC	–5.5	–7.0, –4.1	<0.01	0.05
MQ	1.9	–0.2, 4.1	0.06	0.00
OH-TBZ	–5.5	–7.3, –3.6	<0.01	0.03
OH-PYM	11.9	9.3, 14.4	<0.01	0.08
OH-TEB	12.2	10.5, 14.5	<0.01	0.18
ETU	–3.9	–5.1, –2.7	<0.01	0.04

Annual change (*β*, % per year) in concentrations (ln-transformed) of pesticide biomarkers in urine from Swedish adolescents in Scania, 2000–2017, for all samples.

All the observed trends were highly significant, although the coefficient of determination was very low for all regression models. Biomarkers that failed to meet the assumptions of linear regression, and therefore were not further evaluated, were CFCA, 2,4-D, 4F-3-PBA, and MCPA. Overall, the estimates of the regression models did not markedly change after performing the sensitivity analyses suggesting that the trends were robust (Supplementary VII, Table D).

## Discussion

### Biomarker concentrations in urine

CCC, or chlormequat, displayed the highest median and maximum concentrations among the studied biomarkers. This substance has been extensively used as a growth regulator in grain production and is approved for use in Sweden and in several products in the rest of the EU. CCC is reportedly the most frequently detected pesticide in samples of Swedish rye [46]. Higher concentrations of CCC relative to those of the other biomarkers were therefore not surprising. A national food survey of Swedish adolescents, 2016–2017, shows that secondary school students consume more grain products than vegetables and fruits, lending some support to our findings [47].

TCPy, a metabolite of the organophosphorus insecticides chlorpyrifos and chlorpyrifos-methyl, was overall found in the second highest median concentrations. This result was less expected, as chlorpyrifos has never been approved for plant protection in Sweden. However, this pesticide is allowed in many countries inside and outside the EU. Chlorpyrifos can therefore be connected to the consumption of imported food. Further, the National Food Agency in Sweden has reported that chlorpyrifos occasionally exceeded the maximum residue levels in some imported fruits [4, 36].

The measured concentrations only reflect exposure within the past 24 h and are probably associated with very recent intake of food, since these compounds have short biological half-lives [38, 48]. Furthermore, the absolute measured urinary concentrations of each compound can be difficult to compare because the metabolism and toxicokinetic parameters of the compounds vary.

The population characteristics BMI and smoking habits were comparable between our study population and the general population based on statistics reported by the Public Health Agency of Sweden [49]. Our study population can therefore be deemed representative of the general population of the same age regarding these characteristics. Only males were recruited during the first 3 sampling years. It is plausible that the exposure levels of females might differ from those of males. However, exclusion of females in the sensitivity analysis did not change the estimates markedly, thus suggesting that the time trends are consistent over sex.

There are difficulties comparing the present concentration levels with those reported in other biomonitoring studies because of variability in sample collection procedures, analytical methods, population characteristics, urine dilution correction methods, sampling years, and national differences in commercial use of compounds. Most biomonitoring studies of pesticides in the general population have measured pyrethroid and organophosphate insecticides and phenoxy herbicides. Comparable measurements of the other compounds studied here have, to our knowledge, not been published elsewhere. For pyrethroids, the metabolite 3-PBA has commonly been detected in the highest concentrations in previous studies whereas 4F-3-PBA has mainly been below LOD, which is in line with our results. Generally, the median concentrations of TCPy in our study (0.82–1.41 µg/L) were similar to the reported median among children (0.9 µg/L) and slightly lower than those reported for pregnant women (1.6–3.2 µg/L) in the USA [21, 24]. Furthermore, median concentrations of TCPy found in our study were similar to those found in children residing near banana plantations (1.4–1.6 µg/L) in Costa Rica [12]. Creatinine adjusted concentrations of TCPy and CCC have been reported in a general population, including people residing close to agricultural areas, in the UK [29]. The 95th percentile for TCPy (10.1 µg/g creatinine) were higher in the UK population compared with our study (2.4–5.2 µg/g creatinine). For CCC, reported median (15.1 µg/g creatinine) and 95th percentile (79.8 µg/g creatinine) concentrations were both higher in the UK compared with our population (median; 1.2–3.9 µg/g creatinine, P95; 13.6–66.1 µg/g creatinine).

Median concentrations of pyrethroid biomarker 3-PBA (0.11–0.21 µg/L) in our study were similar to, or slightly lower than, those found in children from the USA aged 12–19 years (0.16–0.30 µg/L), aged 3–5 years (0.46 µg/L), German children (0.29 µg/L), and in the Canadian general population (0.22–0.36 µg/L) during the same period [20, 21, 25, 26]. Median concentrations of 3-PBA in children in Costa Rica (0.7–0.8 µg/L) were higher [12]. The urine concentrations of CFCA at the 95th percentile was lower in our study (0.03–0.4 µg/L) compared with a general population study in the UK (3.2 µg/L). Measured median concentrations of 2,4-D in our study (0.12–0.13 µg/L) were slightly lower than reported in American children 3–5 years (0.21 µg/L) and in the Canadian general population (0.22 µg/L) [21, 26]. The 95th percentile in our population (0.35–0.69 µg/L) was similar to that of pregnant women (0.3–0.6 µg/L) in the USA [24]. For ETU, creatinine adjusted mean concentration in our study (0.3–0.7 µg/g creatinine) were similar to the estimated mean concentration (0.6–0.8 µg/g creatinine) in a group from the general population in Italy [31]. Further, density-adjusted median concentrations of ETU (0.2–0.4 µg/L) were around five times lower in our study than the median concentration (1.2 µg/L) in children in Costa Rica [12].

### Time trends

A general increasing trend in levels of TCPy exposure was observed over time in the linear regression, although the boxplot suggested a decline after 2009. Chlorpyrifos and chlorpyrifos-methyl are partly metabolized to TCPy in the body after dietary intake but TCPy has been confirmed to be present in food products as well, caused by degradation in the fruit or vegetable during food processing [50]. Concentrations measured in urine are therefore often higher than expected relative to chlorpyrifos and chlorpyrifos-methyl concentrations measured in food. However, this would not affect observed trends, because the use of chlorpyrifos in food production is the major source of both the parent compound and the metabolite found in food. The observed increasing trend might, in part, have been caused by the renewed permit for chlorpyrifos, and chlorpyrifos-methyl, in some EU countries in 2006 [51]. In addition, the National Food Agency in Sweden reported high residue levels of chlorpyrifos in some samples of imported apples, carrots, and citrus fruits in its latest publication on food monitoring data from 2016 [4].

For pyrethroids, an increasing trend was observed for the nonspecific biomarker 3-PBA. In contrast, no trend could be seen for DCCA, a specific biomarker of the pyrethroids permethrin, cypermethrin, and cyfluthrin which can also form the nonspecific metabolite 3-PBA (Table 2). The increasing trend of 3-PBA is therefore probably explained by pyrethroid compounds other than those specifically forming DCCA, such as lambda-cyhalothrin used for plant protection in Sweden until 2017 that forms both metabolites 3-PBA and CFCA. Similarly, as for TCPy, the metabolite 3-PBA can also be present as a residue in food due to environmental degradation and direct exposure to 3-PBA is therefore also possible [52].

The fungicides pyrimethanil (OH-PYM) and tebuconazole (OH-TEB) had the highest percentage increase over time (on average >10% per year). Pyrimethanil use is allowed in Sweden with restrictions to certain fruits, but is more extensively used in the EU. Pyrimethanil residues have been detected in both Swedish and imported fruits and other crops in the food monitoring program run by the National Food Agency [4, 35, 36]. Tebuconazole is also allowed in agriculture, mainly for grain production in the EU, including Sweden [51]. Furthermore, pyrimethanil has a different mode of action from that of, for example, thiabendazole and other commonly used fungicides. Fungal resistance to thiabendazole has been reported in the USA, which may contribute to the increased use of pesticides with a different mode of action, such as pyrimethanil [53]. This could be one possible explanation to an increasing trend in the biomarker OH-PYM in urine concentrations in the population.

A decreasing trend was observed for residues of the grain growth regulator CCC. It is the most frequently detected pesticide in Swedish rye, although measurements from the National Food Agency indicate that CCC was detected in 60% of samples in 2013 but only 41% of samples in 2018 [4]. This supports the decreasing trend in CCC biomarkers in urine samples from the population in the present study. In recent years, several other growth regulators have been approved for use, which could explain the decrease in CCC use [46]. The other growth regulator studied here, MQ, was overall found in lower concentrations than CCC. This agrees with measurements in grains made between 1992 and 2017 by the National Food Agency, which frequently detected lower concentrations of MQ than those of CCC [46]. However, MQ can also be formed during food roasting processes and can therefore be detected in products for which it is not registered for use [54].

Decreasing trends were observed for OH-TBZ and ETU, the former being a biomarker for thiabendazole and the latter being a nonspecific biomarker of ethylene bis-dithiocarbamates (EBDCs), mainly mancozeb. Thiabendazole is used in the EU and was permitted for use in Sweden until 2002, when the active substance was banned. However, it is still reported as one of the most common compounds detected in some imported fruits [35, 36]. Still, the national restriction in Sweden could be connected to the decreasing trend. Mancozeb (a common EBDC) is permitted in both Sweden and the EU. The biomarker ETU could be a residue of several EBDCs, but mancozeb is the most commonly used EBDC globally and allowed for use in Sweden. The metabolite ETU can further be formed during the processing of EBDC-treated food, which also contributes to the concentrations detected in urine [55]. It has been suggested that ETU exposure is associated with a wider range of toxic mechanisms in animal studies than exposure to the parent compound mancozeb [56].

Generally, adolescents consume fewer fruits and vegetables than do older population groups, such as middle-aged women [57]. A national food survey of adolescents in Sweden, 2016–2017, further showed that, in secondary school, females consume more fruit and vegetables than do males [47]. Despite this, all initially observed trends in this study remained after the sensitivity analyses excluding females as well as observations above the 95th percentile, respectively. Overall, we can conclude that the observed trends are truly present in the material and not driven by influential observations.

The coefficient of determination (*R*^2^) was low for all regression models, indicating that the variance in concentrations is explained by factors other than calendar year.

Our lab has previously conducted human exposure experiments for CCC [40], OH-PYM [38], OH-TBZ [58], ETU [39], 2,4-D, and MCPA [48] in which volunteers were administered a single oral doses equal to, or 50% of, the accepted daily intake (ADI). The measured urine concentrations from the exposed volunteers in these studies were up to 1000 times higher than those found in Swedish adolescents. Even the maximum concentrations found in the present study’s populations were far below the levels of the exposed volunteers. Although, potential health risks due to cumulative exposure concentrations of multiple pesticides has not been considered here.

The high detection frequency (% >LOD) of pesticide metabolites in this population of adolescents from Sweden 2000–2017 indicates exposure to low levels of pesticides or their breakdown products in adolescents in southern Sweden. It should also be noted that TCPy was found in the second highest concentrations, even though chlorpyrifos has never been registered for plant protection in Sweden.

### Strengths and limitations of the study

This is one of few available studies covering repeated cross-sectional measurements of urinary concentrations of several contemporary pesticides over an extensive period of 17 years in adolescents. It is a large study that includes biological samples from many individuals from the general population. Further, several compounds that rarely have been monitored in the general population were measured. It is also a great advantage that the study population was homogenous in terms of participant ages and geographic area throughout the sample collection, although differences in exposure levels between different age groups and geographic areas must be considered with respect to external validity.

The long-sample storage up to 17 years could impact the quality of the samples and the stability of biomarkers. We have examined the stability for TCPy and 3-PBA in urine samples from a population (*n* = 296), before and after 4 years of storage [unpublished data]. These samples were analyzed using a simplified method. The analyses showed excellent results. This data implies that these two compounds may be stable at −20 °C for at least 4 years. For the samples in the present study we looked at the density in all the samples to see if it differs between the sampling years. We could not see any difference or change in density depending on sampling year in this material.

The main source of pesticide exposure in the general population is through residues in consumed food products [59]. The pesticides examined here are rapidly metabolized and/or excreted [38, 39, 58], so the concentrations represent only recent exposure (within the past 24 h). Our samples consisted of random spot urine samples with no record of what time of day they were collected. A study of pyrethroids showed that 24-h void urine samples generally contained higher concentrations than did the first morning urine samples [60], so spot urine samples may underestimate daily average concentrations. Even with these limitations, it was possible to detect temporal trends of exposure to some of the studied pesticides.

The most common correction method in biomonitoring studies is creatinine adjustment, which assumes that creatinine is excreted in urine at a constant rate. However, several studies have shown that creatinine excretion can vary greatly depending on sex, age, diet, and muscle mass [60]. Data adjusted through both correction methods are presented but density adjustment was preferred in the statistical analysis because both males and females were included. However, both these adjustment methods could affect the individual variability in the observations.

The measured values from all observations were used in the statistical models, including values below LOD. These values have a high level of uncertainty with the “true” value being somewhere between zero and LOD. As the focus was to study concentration trends over time, imputation through fixed substitution methods could dilute the variability in the material [43] affecting the observations of possible trends. The measured values are a better reflection of the true values than fixed substitution methods and were therefore preferred for this aim, even though the higher uncertainty tend to increase the residual variance around the regression line.

The trends were not mainly explained by sampling year, as shown by the low coefficients of determination. Without information on dietary intake, lifestyle factors, and seasonal variations we cannot further comment on whether the observed trends were related to changes in the agricultural use of these compounds, import of food, seasonal variation, or lifestyle factors.

## Conclusions

We found 13 of 14 biomarkers of contemporary pesticides in measurable concentrations in more than 50% of urine samples, indicating widespread exposure. Furthermore, we found that OH-PYM, OH-TEB, 3-PBA, and TCPy were increasing in concentration over time. Surprisingly, the second highest median concentration was found for TCPy even though chlorpyrifos and chlorpyrifos-methyl have never been permitted for agricultural use in Sweden. We also found decreasing trends for the biomarkers OH-TBZ, ETU, and CCC. There is a widespread pesticide exposure from diet in Swedish adolescents, but the concentrations are low and presumably below recommended ADI values.

## Supplementary information

Supplementary I

Supplementary II

Supplementary III

Supplementary IV

Supplementary V

Supplementary VI

Supplementary VII
