# Supplementary material for: Concentrations and temporal trends in pesticide biomarkers in urine of Swedish adolescents, 2000–2017
Source: J Expo Sci Environ Epidemiol. 2020 Feb 24;30(4):756–67. doi: 10.1038/s41370-020-0212-8 (PMC8075908; doi:10.1038/s41370-020-0212-8)
Supplement: Supplementary file 1 — Supplementary I [file 41370_2020_212_MOESM1_ESM.pdf]

Supplement I

Complete name list of pesticide parent compounds, corresponding biomarkers and abbreviation.

Table A. Parent compound, trivial name, name according to IUPAC and abbreviations of corresponding biomarkers.

Parent compound	Biomarker (trivial)	Biomarker (IUPAC)	Abbreviation
**thiabendazole**	5-hydroxy-thiabendazole	5-hydroxy-2-(4-thiazolyl) benzimidazole	OH-TBZ
**pyrimethanil**	4-hydroxy-pyrimethanil	4-[(4,6-dimethyl2-pyrimidinyl) amino] phenol	OH-PYM
**tebuconazole**	hydroxy tebuconazole	5-(4-chlorophenyl)-2,2-dimethyl-3-(1*H*-1,2,4-triazol-1-ylmethyl)-1,3-pentanediol	OH-TEB
**chlorpyrifos**	3,5,6-trichloro-2-pyridinol	3,5,6-trichloro-2-pyridinol	TCPy
**pyrethroids**[1]	3-phenoxybenzoic acid	3-phenoxybenzoic acid	3-PBA
**cyfluthrin**	4-fluoro-3-phenoxybenzoic acid	4-fluoro-3-phenoxybenzoic acid	4F-3-PBA
**permethrin, cypermethrin, cyfluthrin**	3-(2,2-dichlorovinyl)-2,2-dimethylcyclopropanecarboxylic acid	3-(2,2-dichlorovinyl)-2,2-dimethylcyclopropanecarboxylic acid	DCCA
**bifenthrin**	3-(2-chloro-3,3,3-trifluoroprop-1-enyl)-2,2-dimethylcyclopropanecarboxylic acid	(1RS,3RS)-3-((Z)-2-chloro-3,3,3-trifluoroprop-1-enyl)-2,2-dimethylcyclopropanecarboxylic acid	CFCA
**2,4-D**	2,4-dichlorophenoxyacetic acid	2,4-dichlorophenoxyacetic acid	2,4-D
**MCPA**	2-methyl-4-dichlorophenoxyacetic acid	2-methyl-4-chlorophenoxyacetic acid	MCPA
**EBDCs**[2]	ethylene thiourea	Imidazolidine-2-thione	ETU
**propineb**	propylene thiourea	4-methyl-2-imidazolidinethione	PTU
**chlormequat**	chlormequat	chlormequat	CCC
**mepiquat**	mepiquat	mepiquat	MQ
