# Supplementary material for: Concentrations and temporal trends in pesticide biomarkers in urine of Swedish adolescents, 2000–2017
Source: J Expo Sci Environ Epidemiol. 2020 Feb 24;30(4):756–67. doi: 10.1038/s41370-020-0212-8 (PMC8075908; doi:10.1038/s41370-020-0212-8)
Supplement: Supplementary file 2 — Supplementary II [file 41370_2020_212_MOESM2_ESM.pdf]

Supplement II - Analytical method I

The method previously described in Faniband et al (36) was used with some modifications, for analysis of the biomarkers OH-TBZ, OH-PYM, OH-TEB, TCPy, 3-PBA, 4F-3-PBA, DCCA, CFCA, 2,4-D and MCPA in urine samples.

Chemicals and materials

The metabolite OH-TBZ was purchased from Dr Ehrenstorfer (Augsburg, Germany). The metabolite TCPy was purchased from Sigma-Aldrich (Steinhein, Germany) and 2,4-D was from Acros Organics (Geel, Belgium). The metabolite OH-PYM, CFCA and the internal standards [D_4]-OH-PYM, [$^{13}C_2$][^{15}N]-5-OH-TBZ and [$^{13}C_5$]-TCPy were purchased from Toronto Research Chemicals (North York, ON, Canada). The metabolites 4F-3-PBA and DCCA and the internal standards [D_3]-2,4-D, [D_6]-DCCA (cis/trans), [$^{13}C_6$]-3-PBA and [$^{13}C_6$]-4F-3-PBA were purchased from Cambridge Isotope Laboratories, Inc. (Andover, MA, USA). The compound MCPA and metabolite 3-PBA were from Sigma-Aldrich Inc. (St. Louis, MO, USA). The IS [D_3]-MCPA was purchased from QMX Laboratories (Thaxted, UK). The metabolite OH-TEB and IS [D_6]-TEB were kindly provided by Silvia Fustinoni (Fustinoni et al., 2014).

Formic acid (FA) was from Sigma-Aldrich Inc. (St. Louis, MO, USA). Acetic acid (glacial) was from Fisher Scientific (Loughborough, UK). Methanol and acetonitrile (hyper grade for LC-MS) and ammonium acetate (EMSURE ACS, Reag. Ph Eur) were from Merck (Darmstadt, Germany). β-glucuronidase/arylsulfatase from *Helix pomatia* and β-glucuronidase from *E. Coli* were both purchased from Roche Diagnostics Scandinavia AB (Bromma, Sweden). Solid phase extraction (SPE) columns, silica-based ISOLUTE®-96 ENV + 40 mg fixed-well plates were from Biotage (Uppsala, Sweden). Water was produced by Milli-Q Integral 5 system, Millipore (Billerica, MA, USA). The 96-well plates were from Sorbent AB (Västra Frölunda, Sweden).

Instrumentation

Samples were analysed using a triple quadrupole linear ion trap mass spectrometer, equipped with TurboIonSpray source (QTRAP 5500; AB Sciex, Foster City, CA, USA) coupled to a liquid chromatography system (UFLC^{RX}, Shimadzu Corporation, Kyoto, Japan). The analysis was performed by selected reaction monitoring (SRM). Pure nitrogen was used as curtain- and collision gas. Air was used as nebulizer and auxiliary gas and set at the temperature 650°C and the ion spray voltage at 5500 V. All data acquisition was performed using Analyst 1.6.3 software and data processing was performed using Multiquant 2.1 (AB Sciex).

Calibration standards and quality controls (QC)

Parent compounds and/or metabolites were accurately weighed in 10mL flasks in duplicates and dissolved in methanol to prepare stock solutions. The calibration standards were prepared by spiking a blank urine sample (obtained from a healthy volunteer) of 475 µL with 25 µL stock solution, ranging from 0 to 50 µg/L. The quality controls were prepared from blank urine spiked with the analytes. The final concentrations of QCs were 2 µg/L (QC low) and 20 µg/L (QC high). Chemical blanks were prepared in Milli-Q water.

Sample preparation

Urine samples were thawed after storage in freezer at -20ºC and aliquots of 500 µL were pipetted into 2 mL 96-well-plates (Ritter; Sorbent AB, Västra Frölunda, Sweden). Urine samples, calibration standards, quality controls and chemical blanks were added with 150 µl of 1 M ammonium acetate buffer (pH 6.5) 10 µl β-glucuronidase/arylsulfatase enzyme and 25 µl of IS solution. Internal standards were available for all compounds except CFCA (Table A). Plates were covered with silicon mats (Sealing Mat, 96 square-well, Kinesis, Cambridgeshire, UK), vortex mixed and incubated overnight at 37ºC with agitation (400 rpm). The urine samples were then applied on the SPE plates preconditioned with 1 ml MeOH and then 1 ml water. Samples were applied and washed with 1ml water and further with 1% acetic acid in 40% methanol. The analytes were eluted into a 96-well plate (2 ml) with 1 ml acetonitrile. The 96-channel equipment CEREX was used in the extraction. The eluted samples were evaporated at 60ºC for one hour using MiniVap. The analytes were re-dissolved in 80 µl MeOH and

40 µl water per well, mixed and transferred to a new plate with 0.5 ml glass inserts (Sorbent AB, Sweden) and covered with sealing cap mat (Crelab Instruments, Sweden). The samples were mixed and centrifuged at 3000 x g for 10 min before analysis.

Analysis

The chromatographic separation for OH-TBZ, OH-PYM, OH-TEB was carried out on a Poroshell 120EC-C18 column (4.6 x 100 mm, 2.7 µm, Agilent Technologies, Santa Clara, CA, USA). The mobile phase consisted of 0.1 % formic acid in Milli-Q water as (A) and 0.1% formic acid in methanol as (B). The sample injection volume was 3.0 µL and the flow rate through the column was 0.7 mL/min with a column temperature maintained at 40ºC. The mobile phase gradient started with 30 % mobile phase B for 1 min, followed by a linear gradient to 95 % in 3.6 min and held at 95 % for 1.0 min before equilibration at 30 %. The total analytical run time per sample, including equilibration, was 7.0 min. The analysis was performed in the positive mode with electro spray ionization (ESI) and the transitions are showed in Table B, supplement V.

The chromatographic separation for TCPy, 3-PBA, 4F-3-PBA, DCCA, CFCA, 2,4-D and MCPA was carried out on a Genesis Lightn C18 column (2.1 x 100 mm, 4.0 µm, Hichrom limited, Leicestershire, UK). The mobile phase consisted of 0.1% formic acid in Milli-Q water as (A) and 0.1% formic acid in methanol as (B). The sample injection volume was 6 µL and the total flow rate through the column was 0.6 mL/min with a column temperature maintained at 40ºC. The mobile phase gradient started with 5 % of mobile phase B and increased linearly to 60 % in 6 min, followed by an increase to 95 % at 6.6 min and held at 95 % until 7.2 min before equilibration at 5 %. The total analytical run time per sample, including equilibration, was 9 min. The analysis was performed in the negative mode, and the transitions of the analytes are showed in Table A, supplementary.

Each sample batch contained calibration standards, chemical blanks (prepared from Milli-Q water) and quality control (QC) samples. Samples from year 2000 to 2013 were analyzed together (randomized) within 6 months. Samples from year 2017 were analysed 12 months later with the same quality control samples and internal standards. All analyses were performed by the same laboratory technician. The

laboratory took part in the Erlangen inter-laboratory program for TCPy and 3-PBA with excellent results during the analysis period.

Limit of detection and precision

Limit of detection (LOD) was defined as three times the standard deviation of the concentration corresponding to the peak area ratio in the chemical blanks. The mean value of the chemical blanks from all batches (n = 14) of samples was used to estimate the LOD for each biomarker. The precision of the method was determined as between-batch precision. It is presented as a mean value and coefficient of variation (CV) for quality control samples in Table C, supplement VI.
