# Supplementary material for: Concentrations and temporal trends in pesticide biomarkers in urine of Swedish adolescents, 2000–2017
Source: J Expo Sci Environ Epidemiol. 2020 Feb 24;30(4):756–67. doi: 10.1038/s41370-020-0212-8 (PMC8075908; doi:10.1038/s41370-020-0212-8)
Supplement: Supplementary file 3 — Supplementary III [file 41370_2020_212_MOESM3_ESM.pdf]

Supplement III - Analytical method II

The method previously described in Lindh et al. (38) was used with some modifications, for analysis of the biomarkers CCC and MQ in urine samples.

Chemicals and materials

Pure standards of CCC and MQ were purchased from Sigma-Aldrich (St Louis, MO, USA). The internal standard (IS) [D_4]-CCC was purchased from Dr. Ehrenstorfer (Augsburg, Germany) and the IS [D_{16}]-MQ was purchased from C/D/N Isotopes Inc. (Pointe-Claire, Quebec, Canada). Acetonitrile, methanol, ammonium acetate, acetic acid and formic acid were from Merck (Darmstadt, Germany). Water was produced by Milli-Q Integral 5 system, Millipore (Billerica, MA, USA). The solid phase extraction (SPE) columns silica-based ISOLUTE® HCX-Q (100mg) 96-fixed well plates were from Biotage (Uppsala, Sweden). The 96-well plates for sample preparation were from Sorbent AB (Västra Frölunda, Sweden). Water was produced by Milli-Q Integral 5 system, Millipore (Billerica, MA, USA).

Instrumentation

Quantitative analysis was conducted using a triple quadrupole linear ion trap mass spectrometer equipped with a TurboIonSpray source (QTRAP 5500; AB Sciex, Foster City, CA USA) coupled to a liquid chromatography system (UFLC^{XR}, Shimadzu Corporation, Kyoto, Japan). Air was used as nebulizer and auxiliary gas. Pure nitrogen was used as curtain gas and collision gas. The temperature of the auxiliary gas was set at 550°C and the ion spray voltage was 4500 V. The MS analyses were carried out using selected reaction monitoring (SRM) in the positive ionization mode. All data acquisition was performed using Analyst 1.6.3 software and data processing was performed using Multiquant 2.1 (AB Sciex).

Calibration Standards

Stock solutions were prepared in duplicates by dissolving accurately weighed amounts of internal [D_4]-CCC, CCC, [D_{16}]-MQ and MQ in 10 mL water. Standard solutions were further prepared by

dilution of stock solutions in water. The calibration standards were prepared by spiking 100 µL urine with the standard solutions. Urine samples for the calibrations standards and quality control (QC) samples were obtained from healthy volunteers in the laboratory. Urine with a low amount of CCC was selected for the calibration standards. The urine was spiked with 0 - 50 µg/L CCC and then corrected with the amount found in the urine. Two quality control urine samples with quantified levels of 7.0 µg/L and 31 µg/L of CCC were selected and used in all analytical batches. Each analytical batch also included a calibration curve including seven standards levels, a chemical blank and a urinary blank.

Sample preparation

Urine samples, calibration standards, quality controls and chemical blanks were pipetted to a 2 ml 96-deep well plate and thereafter added with 0.9 mL of 0.01 M ammonium acetate (pH 7) containing 1 ng of each IS, [D_4]-CCC and [D_{16}]-MQ. Plates were covered with silicon mats (Sealing Mat, 96 square-well, Kinesis, Cambridgeshire, UK) and vortex-mixed. The samples were extracted by SPE-columns. The columns were preconditioned with 1 mL methanol followed by 1 mL of 0.01 M ammonium acetate. The samples were thereafter applied on the columns followed by washing step of 1 ml 0.01 M ammonium acetate. The samples were then eluted from the columns with 1 % formic acid in 1 mL methanol and transferred to a new 96-deep well-plate. The plates were covered with a polypropylene lid and centrifuged at 3000 x g for 10 minutes immediately before analysis.

Analysis

The chromatographic separation for CCC and MQ was carried out on an Atlantis HILIC column (150 mm x 2.1 mm, 3µm; Waters, Milford, MA, USA). The mobile phase consisted of 0.05 M ammonium acetate buffer (pH 3.75) in Milli-Q water as (A) and acetonitrile as (B). The sample injection volume was 3 µL and the flow rate through the column was 0.3 mL/min. with a column temperature maintained at 40ºC. The mobile phase gradient started with 30% mobile phase B for 1.0 min and thereafter increased linearly up to 95% in 3 min and held for 0.2 min. It was brought back to 30% of mobile phase B and equilibrated at 30 % for 2 min. The total runtime was 5.3 min. The flow was

diverted to waste for the first 3 min. The transitions used in the positive mode with selected reaction monitoring (SRM) are showed in Table B, supplement V.

Each sample batch contained calibration standards, chemical blanks (prepared from Milli-Q water) and quality control (QC) samples. Samples from year 2000 to 2013 were analyzed together (randomized) within 6 months. Samples from year 2017 were analysed 12 months later with the same quality control samples and internal standards. All analyses were performed by the same laboratory technician.

Limit of detection and precision

Limit of detection (LOD) was defined as three times the standard deviation of the concentration corresponding to the peak area ratio in the chemical blanks. The mean value of the chemical blanks from all batches (n = 14) of samples was used to estimate the LOD for each biomarker. The precision of the method was determined as between-batch precision. It is presented as a mean value and coefficient of variation (CV) for quality control samples in Table C, supplement VI.
