# Supplementary material for: Concentrations and temporal trends in pesticide biomarkers in urine of Swedish adolescents, 2000–2017
Source: J Expo Sci Environ Epidemiol. 2020 Feb 24;30(4):756–67. doi: 10.1038/s41370-020-0212-8 (PMC8075908; doi:10.1038/s41370-020-0212-8)
Supplement: Supplementary file 4 — Supplementary IV [file 41370_2020_212_MOESM4_ESM.pdf]

Supplement IV - Analytical method III

The method previously described in Ekman et al (2013) was used with some modifications, for analysis of the biomarkers ETU and PTU in urine samples.

Chemicals and materials

The internal standard (IS) [D_4]-ETU was purchased from Dr. Ehrenstorfer (Augsburg, Germany). ETU and PTU PESTANAL® analytical standards and formic acid (FA) were from Sigma-Aldrich (St. Louis, MO, USA). Sodium hydroxide (NaOH), hydrochloric acid (HCl) and methanol (hyper grade for LC-MS) were from Merck (Darmstadt, Germany).

The 96-well-plates block with clear glass inserts of 1.5 mL and a sealmat block cover (molded PTFE/Silicone Liner) were from Scantec Nordic (Partille, Sweden). The 96-well-aluminium plates with 1.5 mL aluminium block cover, was from J.G. Finneran Associates, Ltd (Surrey, United Kingdom).

Instrumentation

Quantitative analysis was conducted using a triple quadrupole linear ion trap mass spectrometer, equipped with TurboIonSpray source (QTRAP 6500+; AB Sciex, Foster City, CA, USA) coupled to a liquid chromatography system with four pumps (UFLC^{XR}, Shimadzu, Corporation, Kyoto, Japan). Air was used as nebulizer spraying gas and pure nitrogen was used as curtain gas. All data acquisition was performed using Analyst 1.6.3 software and data processing was performed using Multiquant 2.1 (AB Sciex).

Calibration standards and quality controls (QC)

Stock solutions were prepared by dissolving accurately weighed amounts of [D_4-ETU], ETU and PTU. No internal standard (IS) was available for PTU. The IS, ETU and PTU standard stock solutions were further diluted in methanol. Urine samples for the calibration standards and for quality control samples were obtained from healthy volunteers at our laboratory. For the calibration curve, 475 µl blank urine was spiked with 25 µl of the IS solution, giving a urinary concentration for ETU and PTU between 0

and 40 µg/L and 5 µg [D_4-ETU]/L urine. The calibration curve was corrected with the amount found in the urine. For quality control (QC), a urine sample naturally containing 4 µg ETU/L urine (but no PTU) was spiked with 2 µg ETU + PTU/L urine (QC low) and 20 µg ETU + PTU/L urine (QC high) were prepared. This gave a final concentration of approximately 6 µg ETU/L and 2 µg PTU/L in QC low and 24 µg ETU/L and 20 µg PTU/L in QC high. The chemical blank was prepared from Milli-Q water and thereafter treated like the other samples.

Sample preparation

Aliquots of 500 µl of urine samples and QC-samples were transferred into 1.5 mL glass vials and placed in an aluminium 96-well plate. Thereafter, 25 µl of IS solution was added. For the hydrolysis, 20 µl of 2.5 M NaOH was added to the samples, standards, QC-samples and chemical blanks. To prevent evaporation during hydrolysis, the glass vials were sealed with a sealmat and a lid. After sealing, the samples were mixed thoroughly for 15 seconds and then transferred to a heating oven. Hydrolysis was performed for 1 h at 100 °C. After hydrolysis, the samples were cooled to room temperature and the glass vials were moved to new plastic 96-well-plates (Scantec Nordic). Aliquots of 15 µl of 5 M HCl were added to acidity the samples. The samples were mixed for 15 seconds and then centrifuged for 10 min at 2600 x g before analysis.

Analysis of ETU

The mobile phases used consisted of 0.1 % FA in Milli-Q water (A) and 0.1% FA in methanol (B). The two-dimensional separation of ETU was carried out using the analytical columns Fortis H2O (100 x 4.6 mm, 3 µm) and Restek Ultra AQ (100 x 4.6 mm, 3 µm) and two sets of LC pumps. The columns and LC pumps were connected through a diverter valve. An aliquot of 20 µL of the sample was injected on the first column and the separation was carried out by isocratic elution, using 98 % mobile phase A and a flow rate of 0.7 mL/min. After 9 min, the diverter valve was switched over and the effluent was diverted into the second column during 0.5 min and thereafter the diverter valve switched over again. The second set of pumps continued the isocratic elution of the analytes on the second column, using 98 % mobile phase A. A gradient on the second column was applied between 3.5 – 6.0 min from 2% to 95% mobile phase B followed by 1 min at 5 % mobile phase B. A diverter valve on

the MS diverted the column effluent to the MS between 5.8 – 7.1 min. The first column was cleaned with 95% mobile phase B at a flow rate of 1.2 mL/min for 1 min, followed by equilibration with 98 % mobile phase A for 2.5 min, during the time ETU was eluting on the second column. The second column was reconditioned with 95% mobile phase B for 0.5 min in the end of the analytical run and then equilibrated with 98 % mobile phase A for 2.5 min in the beginning of the next analytical run. The columns were maintained at 50°C and the total analytical run-time was 7.0 min. The LC-MS/MS analysis was performed using SRM transition in a positive ionisation mode. The temperature of the auxiliary gas was set at 700°C and the ion spray voltage was 3000 V. Concentrations were determined by peak area ratios between analyte and the IS.

Analysis of PTU

The mobile phases consisted of 0.1 % FA in Milli-Q water (A) and 0.1% FA in methanol (B). The two-dimensional separation of PTU was performed on the analytical columns Genesis Lightn C18 (50 x 2.1 mm, 4 µm) and Restek Ultra AQ (100 x 4.6 mm, 3 µm) and two sets of LC pumps. The columns and LC pumps were connected through a diverter valve. An aliquot of 20 µL of the sample was injected on the first column and the separation was carried out by isocratic elution, using 98 % mobile phase A and a flow rate of 0.7 mL/min. After 0.55 min, the diverter valve was switched over and the unretained PTU was diverted into the second column during 0.5 min. The second set of pumps continued the isocratic elution of the analytes on the second column, using 98 % mobile phase A.A gradient on the second column was applied between 2.6 – 5.0 min from 2% to 95% mobile phase B followed by 1 min at 5% mobile phase B. A diverter valve on the MS diverted the column effluent to the MS between 5.2– 6.1 min. The first column was cleaned with 95% mobile phase B at a flow rate of 1.2 mL/min for 1 min, followed by equilibration with 98 % mobile phase A for 2.5 min, during the time PTU was eluting on the second column. The second column was reconditioned with 95% mobile phase B for 0.9 min in the end of the analytical run and then equilibrated with 98 % mobile phase A for 2.5 min in the beginning of the next analytical run. The columns were maintained at 50 °C and the total analytical run-time was 7.5 min. The LC-MS/MS analysis was performed using SRM transition in a positive ionisation mode. No IS was available for PTU and therefore, concentrations were

determined by comparison of two fragments of PTU (Table B, supplement V). PTU concentrations were only reported if the ratio of the fragments were consistent with the standards (± 20 % and > 0.1 µg/L).

Limit of detection and precision

Limit of detection (LOD) was defined as three times the standard deviation of the concentration corresponding to the peak area ratio in the chemical blanks. The mean value of the chemical blanks from all batches (n = 14) of samples was used to estimate the LOD for each biomarker. The precision of the method was determined as between-batch precision. It is presented as a mean value and coefficient of variation (CV) for quality control samples in Table C, supplement VI.
