# Supplementary material for: Concentrations and temporal trends in pesticide biomarkers in urine of Swedish adolescents, 2000–2017
Source: J Expo Sci Environ Epidemiol. 2020 Feb 24;30(4):756–67. doi: 10.1038/s41370-020-0212-8 (PMC8075908; doi:10.1038/s41370-020-0212-8)
Supplement: Supplementary file 5 — Supplementary V [file 41370_2020_212_MOESM5_ESM.pdf]

Supplementary material V

Table B

Quantifier ions, internal standards and ionization mode applied in analytical methods. Internal standard for PTU was not available.

Compound	Ionization mode	Quantifier ions	IS	Quantifier ions (IS)
**TCPy**	negative	195.7/35.0	$^{13}C_5$-TCPy	200.7/35.0
**3-PBA**	negative	213.0/93.0	$^{13}C_6$-3-PBA	219.1/99.0
**4F-3-PBA**	negative	231.1/93.0	$^{13}C_6$-4F-3-PBA	237.1/98.9
**DCCA**	negative	207.0/35.0	D_6-DCCA	213.0/35.0
**CFCA**	negative	241.0/205.0	$^{13}C_6$-3-PBA	219.1/99.0
**2,4-D**	negative	219.1/161.0	D_3-2,4-D	224.1/166.0
**MCPA**	negative	199.1/140.8	D_3-MCPA	204.1/146
**OH-TBZ**	positive	218.0/191.2	[$^{13}C_2$][^{15}N]-5-OH-TBZ	221.0/194.0
**OH-PYM**	positive	216.1/107.0	D_4-OH-PYM	220.1/111.0
**OH-TEB**	positive	324.2/124.9	D_6-TEB	314.2/72.0
**ETU**	positive	103.1/60.0	[$^2H_4$]-ETU	107.1/48.0
**PTU**	positive	117.1/58.1	NA	NA
		117.1/60.1		
**CCC**	positive	122.2/58.1	D_4-CCC	126.2/58.1
**MQ**	positive	114.3/98.3	D_{16} –MQ	130.0/110.0
