# Supplementary material for: Concentrations and temporal trends in pesticide biomarkers in urine of Swedish adolescents, 2000–2017
Source: J Expo Sci Environ Epidemiol. 2020 Feb 24;30(4):756–67. doi: 10.1038/s41370-020-0212-8 (PMC8075908; doi:10.1038/s41370-020-0212-8)
Supplement: Supplementary file 6 — Supplementary VI [file 41370_2020_212_MOESM6_ESM.pdf]

Supplementary material VI

Table C

The between-run precision of the methods presented as mean value and coefficient of variation in the two quality controls spiked with a low or a high concentration of the analyte (QC low and QC high).

Abbreviation	LOD	Mean, QC low	CV, QC low (%)	Mean, QC high	CV, QC high (%)
**TCPy**	0.063	3.79	7.6	23.5	4.9
**3-PBA**	0.009	2.27	13	20.5	10
**4F-3-PBA**	0.005	2.06	5.7	20.9	6.0
**DCCA**	0.017	2.32	11	22.7	9.8
**CFCA**	0.006	2.13	11	21.8	8.1
**2,4-D**	0.108	2.43	8.1	22.2	6.8
**MCPA**	0.016	2.15	7.3	21.9	4.5
**OH-TBZ**	0.002	2.10	4.0	21.1	3.9
**OH-PYM**	0.004	2.30	10	21.7	7.3
**OH-TEB**	0.016	2.11	6.8	20.7	4.2
**ETU**	0.016	6.33	3.2	24.2	3.1
**PTU**	0.10	2.27	22	21.3	22
**CCC**	0.010	8.02	4.7	31.3	4.7
**MQ**	0.011	6.28	4.9	8.40	5.1
