# Supplementary material for: Concentrations and temporal trends in pesticide biomarkers in urine of Swedish adolescents, 2000–2017
Source: J Expo Sci Environ Epidemiol. 2020 Feb 24;30(4):756–67. doi: 10.1038/s41370-020-0212-8 (PMC8075908; doi:10.1038/s41370-020-0212-8)
Supplement: Supplementary file 7 — Supplementary VII [file 41370_2020_212_MOESM7_ESM.pdf]

Supplement VII

Table D

Sensitivity analyses of linear regression model by excluding 1) observations above the 95th percentile and 2) females.

	1) >95th percentile excluded				2) Females excluded (year 2013 – 2017)			
**Biomarker**	***β*** **(%)**	**95 CI (%)**	***p***	***R^2***	***β*** **(%)**	**95 CI (%)**	***p***	***R^2***
OH-TBZ	−4.9	−6.5, −3.2	<0.01	0.03	−7.0	−9.2, −4.8	<0.01	0.04
OH-PYM	9.8	7.5, 12.1	<0.01	0.07	11.0	7.9, 14.2	<0.01	0.06
OH-TEB	11.2	9.7, 12.7	<0.01	0.19	12.0	10.0, 14.1	<0.01	0.15
TCPy	1.11	0.3, 1.9	<0.01	0.01	1.6	0.6, 2.7	<0.01	0.01
3-PBA	3.3	2.3, 4.1	<0.01	0.05	2.3	1.1, 3.7	<0.01	0.08
DCCA	0.3	−0.5, 1.0	0.53	0.00	−0.3	−1.4, 0.8	0.55	0.00
ETU	−3.4	−4.6, -2.3	<0.01	0.03	−4.0	−5.4, −2.5	<0.01	0.03
CCC	−4.8	−6.1, −3.4	<0.01	0.05	−5.1	−6.8, −3.2	<0.01	0.03
MQ	1.3	−0.8, 3.4	0.23	0.00	3.3	0.6, 5.9	<0.05	0.01
